# Hippo Pathway and YAP Signaling Alterations in Squamous Cancer of the Head and Neck

**DOI:** 10.3390/jcm8122131

**Published:** 2019-12-03

**Authors:** Karla Santos-de-Frutos, Carmen Segrelles, Corina Lorz

**Affiliations:** 1 Molecular Oncology Unit, CIEMAT (ed 70A), Ave Complutense 40, 28040 Madrid, Spain; Karla.Santos@externos.ciemat.es (K.S.-d.-F.); carmen.segrelles@ciemat.es (C.S.); 2 Molecular Oncology, Research Institute 12 de Octubre i+12, University Hospital 12 de Octubre, Ave Córdoba s/n, 28041 Madrid, Spain; 3 Centro de Investigación Biomédica en Red de Cáncer (CIBERONC), Ave Monforte de Lemos 3-5, 28029 Madrid, Spain

**Keywords:** head and neck cancer, squamous cancer, Hippo-YAP pathway, YAP, TAZ

## Abstract

Head and neck cancer affects the upper aerodigestive tract and is the sixth leading cancer worldwide by incidence and the seventh by cause of death. Despite significant advances in surgery and chemotherapy, molecularly targeted therapeutic options for this type of cancer are scarce and long term survival rates remain low. Recently, comprehensive genomic studies have highlighted the most commonly altered genes and signaling pathways in this cancer. The Hippo-YAP pathway has been identified as a key oncogenic pathway in multiple tumors. Expression of genes controlled by the Hippo downstream transcriptional coactivators YAP (Yes-associated protein 1) and TAZ (WWTR1, WW domain containing transcription regulator 1) is widely deregulated in human cancer including head and neck squamous cell carcinoma (HNSCC). Interestingly, YAP/TAZ signaling might not be as essential for the normal homeostasis of adult tissues as for oncogenic growth, altogether making the pathway an amenable therapeutic target in cancer. Recent advances in the role of Hippo-YAP pathway in HNSCC have provided evidence that genetic alterations frequent in this type of cancer such as *PIK3CA* (phosphatidylinositide 3-kinase catalytic subunit alpha) overexpression or FAT1 (FAT atypical cadherin 1) functional loss can result in YAP activation. We discuss current therapeutic options targeting this pathway which are currently in use for other tumor types.

## 1. Introduction

Head and neck cancer is a high-incidence poor-prognosis tumor for which molecularly targeted therapeutic options are limited. This has a detrimental impact on the overall survival of patients. Part of the problem is attributable to a significant delay in the molecular characterization of this type of cancer compared to others. However, over recent years, comprehensive studies including genomic, transcriptomic and clinical data of head and neck tumors have identified the most commonly altered genes and signaling pathways in this cancer [1,2,3,4]. They have also established the existence of different molecular subtypes based on transcriptomics [3,5,6,7], and gene expression signatures associated with poor outcome or drug resistance have been reported [8,9]. At present, we need to make an effort to translate this knowledge into the identification of actionable molecules and pathways, that is, clinically and druggable relevant targets that help to broaden and guide head and neck cancer therapeutics. Recently, the Hippo-YAP pathway has been identified as a relevant oncogenic signaling pathway altered across a wide variety of tumor types [10] including head and neck cancer [11]. YAP (Yes-associated protein 1) is the main downstream effector of the pathway and acts as a transcription cofactor regulating the expression of genes involved in cell proliferation, pro-survival and cell migration signals, all of which contribute to the pro-tumorigenic phenotype [12]. Recent evidence shows that some types of tumors could rely on YAP transcriptional regulation [13,14]. This opens the way for strategies targeting YAP as a new therapeutic option for the treatment of cancer. 

## 2. Head and Neck Cancer: Today´s Problems and Needs

Head and neck cancer arises in the upper aerodigestive tract (lips, oral cavity, salivary glands, larynx, nasopharynx, hypopharynx and oropharynx), and is the sixth most common cancer type worldwide and the seventh by cause of death [15]. Current figures are discouraging—nearly 900,000 people are diagnosed with this type of cancer every year [15] and only about half of them will survive the first five years [16]. Most of these tumors are head and neck squamous cell carcinomas (HNSCCs) that develop in the outer layer of the skin and in the mucous membranes of the tract. The specific locations affected by HNSCC are the oral (including lips) and nasal cavities, paranasal sinuses, pharynx and larynx. Classical risk factors for HNSCC are smoking and heavy alcohol consumption. Moreover, patients with genetically inherited diseases such as Li-Fraumeni or Fanconi anemia show increased susceptibility to the development of this type of cancer. However, the etiology of this disease is gradually changing in the Western world where infection with high-risk human papillomavirus (HPV) is the cause of a rising number of these tumors despite reduction in cigarette smoking rates [17]. HPV positive tumors particularly affect the oropharynx and show a better prognosis. They differ from HPV negative tumors not only in their etiology, localization and prognosis, but also in their molecular characteristics [3,18,19,20]. Tumors with HPV infection do not display mutation of classical tumor suppressors and gene expression patterns reveal that they constitute a different subtype within HNSCC [3].

### 2.1. Molecular Alterations in HNSCC

Inactivation of the p53 pathway is a widespread molecular event in HNSCC. Among HPV negative tumors, 92% of cases present an inactivation (mutation or deletion) of the tumor suppressors *TP53* (tumor protein 53, p53) and/or *CDKN2A* (which encodes for p16 and p14arf) [3]. In HPV positive tumors, p53 pathway inactivation is achieved by the viral oncoproteins E6 and E7 [21]. However, therapeutic strategies aimed to reactivate p53 function are not yet available in the clinical setting.

Signaling pathways regulated by growth factors, such as EGFR (epidermal growth factor receptor) and PI3K/AKT (phosphatidylinositide 3-kinase; v-akt murine thymoma viral oncogene homolog), are frequently affected in HNSCC. Both pathways are interconnected and promote cell survival and proliferation, PI3K/AKT/mTOR being the most commonly altered in HNSCC [3,22]. Within this pathway, the *PIK3CA* (phosphatidylinositide 3-kinase catalytic subunit alpha) gene, which codes for the p110α catalytic subunit of PI3K, is the main oncogene in human cancer, conferring cells growth advantage, evasion of apoptosis and invasion capacities [21,23]. Activating mutations of *PIK3CA* have been found in approximately 20% of HNSCC, and increase in *PIK3CA* copy number and/or overexpression is present in up to 40% of the cases [3]. Overexpression of the *PIK3CA* gene is a poor prognosis factor in HNSCC and is associated with the activation of YAP [24]. In contrast to other tumors, mutations in *EGFR* are not frequent in HNSCC (≤5%) [3]. Instead, an increase in copy number and/or expression of the gene has been associated with poor prognosis, metastasis and resistance to radio and chemotherapy [25]. EGFR is the target of the monoclonal antibody Cetuximab, the only growth factor-specific targeted therapy currently used for the treatment of HNSCC [26]. 

Alteration of the cadherin-like protein tumor suppressor *FAT1* (FAT atypical cadherin 1) is a recurrent event (>10%) in human cancer [10]. Across the different cancers sequenced by The Cancer Gene Atlas (TCGA) Consortium, HNSCC is the tumor type that bears the highest rate of alterations in this gene. More than 25% of HNSCC tumors bear *FAT1* mutation or deletion, approximately twice the frequency of *EGFR* alteration in this cancer type [27]. Despite these facts, the molecular mechanisms that contribute to tumor development in the context of loss of FAT1 function are poorly understood. Recently, FAT1 has been identified as a Hippo pathway regulator in HNSCC [27]. Loss of FAT1 hampers the formation of the multimeric Hippo signaling complex leading to unrestrained YAP activity and tumor progression. Thus, YAP and its regulation may be a neglected therapeutic option for HNSCC.

### 2.2. Current Therapies in HNSCC

Existing therapeutic efforts to treat HNSCC include surgery, radiation, chemotherapy and combinations thereof. Despite significant advances, mainly in surgery and radiation procedures, long term survival rates remain alarmingly low and most of the patients who experience recurrent or metastatic disease die within a year of diagnosis [28]. The chemotherapeutic arsenal available to treat this cancer is insufficient and is based on the use of drugs that widely target DNA (Cisplatin, Fluorouracil) or microtubules (Docetaxel, Paclitaxel). To date there are only two molecularly-based treatments approved for HNSCC, the abovementioned anti-EGFR antibody Cetuximab, and the monoclonal anti-PD-L1 (programmed cell death 1 ligand 1) receptor antibodies Pembrolizumab and Nivolumab. Cetuximab was approved for the treatment of HNSCC in 2006 [29], and over time the figures show that the survival improvement of this therapy is modest and only a small group of patients show long-term benefit [26]. It took a decade for the next targeted anti-cancer therapy to come into play. Immune checkpoint inhibitors were introduced in 2016 to treat HNSCC [30,31]; however, long-term solid evidence regarding the benefit of this therapy is still lacking. One of the main problems in the field of targeted therapies in HNSCC is the complete lack of biomarker-based patient selection to allow stratification into subgroups with different therapeutic options, even for the aforementioned molecularly based therapies. The presence of high-risk HPV in oropharyngeal cancer is the only molecular marker currently used for risk stratification [32]. 

New approaches specifically targeting critical molecular pathways are needed to overcome low survival rates in HNSCC. Along these lines, EGFR and PI3K/AKT/mTOR pathway inhibitors in clinical use for other tumor types, such as Erlotinib and Afinitor, are under preclinical evaluation in HNSCC cancer. Furthermore, the specific class I PI3K p110α catalytic subunit inhibitor Alpelisib, that just gained FDA approval for the treatment of breast cancer patients with mutations in *PIK3CA* [33], the gene coding for this protein, is under preclinical evaluation in HNSCC. However, no *PIK3CA* mutation-based patient stratification is being considered in clinical trials with HNSCC patients at this point. 

## 3. Hippo Pathway and YAP Signaling

Hippo-YAP is one of the canonical oncogenic signaling pathways recently analyzed within the framework of the TCGA PanCancer Atlas initiative, which covers >9000 samples from 33 cancer types [10]. A major role for this pathway in the control of cell growth and organ size was uncovered more than a decade ago with the molecular and phenotypical characterization of the Hippo gene in Drosophila melanogaster [34,35,36]. The biological relevance of the pathway is highlighted by the fact that it is evolutionarily conserved from flies to mammals, where orthologs for the different components of the Hippo pathway have been described [37].

**Figure 1:**
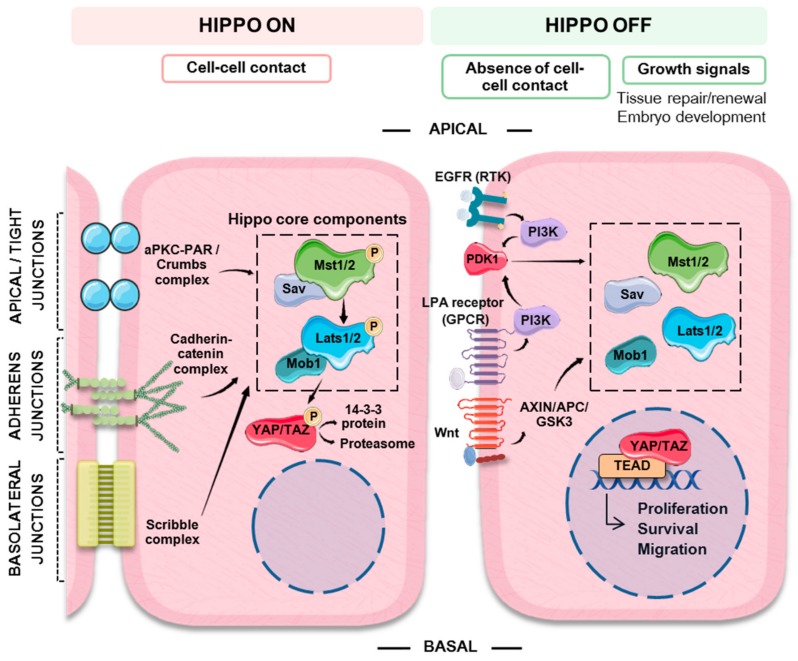
Schematic representation summarizing the main extrinsic (cell–cell contact and growth factors) signals that regulate the Hippo-YAP (Yes-associated protein) pathway. The Hippo-YAP pathway works as a cellular switch. Cell–cell contact promotes the activation of the MST/LATS (mammalian STE20-like protein kinase/large tumor suppressor kinase) core Hippo kinases by different signaling complexes that typically act as scaffolds promoting their phosphorylation. In turn, activated LATS phosphorylates YAP, which is then targeted to ubiquitin mediated proteosomal degradation or to cytosolic sequestration by binding to 14-3-3 protein, thus preventing its nuclear translocation and switching off the expression of its target genes. In the absence of cell–cell contact or in the presence of growth signals, the components of the Hippo core complex are not active. Non-phosphorylated YAP can translocate to the cytosol binding to TEAD (TEA domain transcription factor) family transcription factors (among others) and switching on the expression of genes involved in cell proliferation, survival and migration.

Working as a switch, the multimeric core complex of the Hippo pathway integrates signaling from different upstream cues to control the activity of a downstream effector nuclear transcriptional module (Figure 1). This nuclear module activates the transcription of genes involved in cell proliferation and survival. In fully differentiated tissues and organs, cell–cell contact inhibition works to restrain cell growth. Under these circumstances, the Hippo pathway is usually active (Hippo ON) and the serine/threonine kinases that make up the Hippo upstream core complex phosphorylate and sequester the downstream transcriptional effectors in the cytosol, thus hampering the transcription of target genes. In the absence of cell–cell contact or under cell-growth requiring physiological situations, such as normal tissue repair and renewal or embryo development, the mechanisms blocking gene transcription can be switched off, leading to the activation of genes involved in cell proliferation and survival (Hippo OFF).

### 3.1. Components of the Hippo Pathway

As mentioned above, the Hippo pathway is composed of two main elements, one responsible for the integration of the different stimuli which then controls the activity of a downstream effector element. In mammalians, the cytosolic multimeric signaling complex is formed by the serine and threonine kinases MST1 and MST2 (mammalian STE20-like protein kinase 1 and 2, also STK4 and 3), and LATS1 and LATS2 (large tumor suppressor kinase 1 and 2). MST1/2 and LAST1/2 interact with the adaptor proteins SAV1 (salvador family WW domain containing protein 1) and MOB1 (MOB kinase activator 1), respectively [38,39]. When the Hippo pathway is active, the Drosophila Hippo ortholog MST1/2 phosphorylates and activates LATS1/2, which in turn phosphorylates the transcriptional coactivators YAP and its dimerizing partner TAZ. The approved gene symbol for YAP in humans is YAP1 and is expressed in two isoforms, YAP1-1 and YAP1-2. TAZ is the transcriptional co-activator with a PDZ binding domain; also known as WWTR1, WW domain containing transcription regulator 1 [40,41,42]. YAP1-1, YAP1-2 and TAZ are similar in their structure (TAZ is a paralog of YAP, arising from the duplication of a common gene), consequently they might retain similar functions and many aspects of their regulation are shared. These factors differ mainly in the number of WW domains responsible for binding to regulatory proteins such as LATS1/2 and AMOT, or downstream transcription factors such as RUNX1/2 (Runt-related transcription factor 1 and 2) or TBX5 (T-box transcription factor X5) [43,44], and in the number of residues phosphorylated by LATS1/2 kinases (five in YAP and four in TAZ [40,41]). Additionally, YAP contains sites that can be phosphorylated by c-Abl/Scr/Yes and by JNK kinases or methylated by Set7 histone methyltransferases that are not present in TAZ [45,46,47]. These differences could account for some of the particularities observed in the regulation and/or functions of these factors in certain tissues or contexts [48,49]. 

### 3.2. YAP Signaling

The phosphorylation of YAP and TAZ by LATS1/2 kinases of the Hippo pathway core complex promotes YAP/TAZ interaction with 14-3-3 proteins and their retention in the cytosol, or YAP/TAZ ubiquitination and subsequent degradation [50,51,52]. Either mechanism causes YAP/TAZ nuclear exclusion which impedes the transcription of target genes. Canonical targets of this pathway include genes involved in cell adhesion and epithelial to mesenchymal transition (EMT), development, cell cycle regulation, survival and stemness (*GTGF*, *CYR61*, *COL4A3*, *ITGB2*, *CCNE2*, *CDK2*, *BIRC5* and *SOX9* among others) [53,54]. The largest YAP/TAZ regulated gene transcription signature was accomplished using oral squamous cell carcinoma (OSCC) cell lines [54]. This signature revealed that, at least in this tumor type, YAP has a more prominent transcriptional role than TAZ. It also revealed that YAP canonical targets such as *CTGF* (connective tissue growth factor) or *CYR61* (cysteine rich angiogenic inducer 61) do not show significant changes in their expression with respect to tumor grade or stage [54] (Table 1).

In the absence of MST1/2-LATS1/2 activation, YAP and TAZ are able to translocate to the nucleus where they need to associate with DNA-binding transcription factors to regulate gene expression, since they cannot bind directly to DNA [55]. The main transcription factors mediating YAP/TAZ activity are those belonging to the TEA domain transcription factor (TEAD) family, comprised of TEAD1 to TEAD4. In fact, YAP function can be abolished in the absence of TEADs or if the YAP-TEAD interaction is impaired [56,57]. YAP and TAZ can bind to transcription factors other than TEADs, such as AP-1 (activator protein 1), the intracellular domain (ICD) of ERBB4 (erb-b2 receptor tyrosine kinase 4), Smads, transcription factors of the RUNX family and p73. Binding to these factors can occur in YAP-TAZ-TEAD complexes, thus further modulating transcriptional activity. It has been reported that the association of YAP-TAZ-TEAD with AP-1 activates genes involved in S-phase entry in epithelial cells [14]. Similarly, the ICD of ERBB4 interacts with YAP and TEAD to promote migration in breast cancer cell lines [58]. More recently, the discovery of the interaction of the coactivator bromodomain-containing protein (BRD4) with YAP-TAZ-TEAD1 to enhance the transcription of cancer related genes opened the way for new therapeutic strategies targeting BRD proteins to inhibit YAP activity in tumors [13]. Binding of YAP/TAZ to Smads links Hippo and transforming growth factor-β (TGF-β) pathways, thus connecting processes such as cell density to responsiveness to TGF-β [59]. It is worth mentioning that in some tumors, such as gastric and breast cancer, RUNX factors have a tumor suppressor activity through the formation of RUNX-YAP-TEAD complexes that reduce the transcription of YAP-TEAD target genes, thus abolishing YAP oncogenic activity [60,61]. A tumor suppressor role for YAP has been reported. In response to DNA damaging drugs YAP can translocate to the nucleus and interact with p73 promoting its stabilization and subsequent acetylation by p300, this leads to the transcription of p73 proapoptotic target genes p53AIP1 and BAX [62]. Furthermore, in HNSCC cell lines, overexpression of ΔNp63 repressed YAP expression as well as expression of other apoptotic genes promoting cell survival, whereas YAP silencing in this context enhanced proliferation, survival, migration and resistance to cisplatin [63]. These data are in discrepancy with those obtained in OSCC cell lines, showing that YAP silencing or inhibition of YAP phosphorylation and its subsequent degradation promote cell growth, migration and tumorigenesis in in vivo models [54].

### 3.3. Switching the Hippo Pathway ON and OFF

The main signal responsible for the activation of the Hippo pathway is contact with neighboring cells. In epithelial cells, the mechanisms involved in establishing cell orientation (apical, basal and lateral polarity) are important regulators of the pathway. Additionally, cells can “sense” the characteristics of the extracellular matrix and the presence of extracellular soluble growth factors. Multimeric complexes situated in different locations of the cell membrane are responsible for maintaining adherens and tight junctions as well as cell polarity. Strict specification of apical-basolateral polarity is particularly important in epithelial cells, where it regulates essential features such as stemness, differentiation and cell function. In fact, loss of cell polarity leads to dysplasia and eventually to EMT, a hallmark of cancer [64]. The cadherin-catenin complex at adherens junctions, the aPKC-PAR complex at thigh junctions, the apical Crumbs complex and the basolateral Scribble complex [53,59,65], can work as scaffolds that recruit the kinases of the multimeric core complex of the Hippo pathway, promoting the activation of MST1/2 and LATS1/2 and the subsequent inactivation of YAP/TAZ. Furthermore, α-catenin at adherens junctions can sequester phospho-YAP/14-3-3 complexes, directly preventing YAP activation [66] (Figure 1). 

Through regulation of the Hippo-YAP pathway transcriptional targets the cell can respond and adapt to changes of cell density and polarity. The same is true for the extracellular matrix (ECM). The properties of the ECM can vary during physiological processes and disease, such as tissue remodeling and regeneration, inflammation, fibrosis and cancer. Under these circumstances YAP/TAZ transcriptional activity can be modulated. For instance, when different cell types, including human mammary epithelial cells, are grown on stiff or fibronectin rich matrixes, similar to a tumor-associated ECM, the nuclear localization of YAP/TAZ and the transcription of their target genes is promoted; while cells cultured in a soft matrix display cytosolic YAP/TAZ. These experiments were performed in 2D and 3D models including fibronectin-coated glass slides, hydrogels and cells grown on rigid or elastic pillars (microposts) [67]. Using mammary epithelial cells grown on coverslips coated with different surfaces (fibronectin, poly-D-lysine or laminin), Kim et al. showed that integrin receptors on the cell surface bind to fibronectin in the ECM promoting LATS1/2 inactivation through a FAK (focal adhesion kinase)-Src-PI3K-PDK1 (phosphoinositide-dependent kinase 1) kinase cascade [68]. YAP target genes include genes encoding ECM components and ECM-modifying enzymes that alter the ECM composition [53,54]. In turn, the stiffening of the ECM also affects the cancer associated fibroblasts, favoring the deposition of thick and rigid collagen fibers that further sustain proliferation of the cancer cells [69]. Additionally, intracellular cell shape and tension impact on cytoskeleton contractility and regulate the activity of YAP/TAZ through Rho GTPases-Rho associated kinases (ROCK) independently of Hippo core kinases [70]. 

Some elements of the Hippo-YAP pathway are shared with other pathways thus mediating cross-talk with inputs coming from TGF-β, Wnt and growth factor signaling pathways, and metabolism. YAP/TAZ interact with the TGF-β pathway so that responsiveness to TGF-β can be modulated by cell density. The above-mentioned Crumbs complex transmits cell density information by promoting YAP/TAZ cytoplasmic retention. This can suppress TGF-β signaling since TAZ functions as a SMAD nuclear retention factor. Loss of cell density/polarity would cause the disruption of the Crumbs complex and YAP/TAZ nuclear translocation, enhancing TGF-β signaling and predisposing the cells to TGF-β-mediated EMT [59]. Mitogenic signaling factors, such as EGF (epidermal growth factor) and Wnt ligands (Wnts), can oppose the effects of cell-contact growth inhibition and promote YAP/TAZ transcriptional activity [71,72]. The binding of EGF to its receptor EGFR or LPA (lysophosphatidic acid) to G protein-coupled receptors (GPCRs) activates the PI3K-PDK1 axis. In this context PDK1, which forms a complex with MST and LATS kinases favoring LATS1/2 activation, is recruited to the cytoplasmic membrane causing the dissociation of the complex resulting in loss of LATS1/2 activation and nuclear accumulation of YAP [72]. Wnts bind to GPCRs and can induce YAP activation mainly through the Wnt canonical pathway, which involves destabilization of the Axin/APC/GSK3 (axin/adenomatous polyposis coli/glycogen synthase kinase 3) destruction complex and the release of β-catenin and YAP from this complex and allowing the transcription of their target genes [71] (Figure 1). In turn, the Hippo pathway can inhibit Wnt signaling. Cytosolic YAP/TAZ in combination with DVL (dishevelled segment polarity protein) can regulate the stability of β-catenin in the cytosol counteracting Wnt signaling [73,74], for instance high levels of cytosolic YAP inhibit intestinal crypt proliferation [74]. On the contrary, YAP is required for the development of APC-deficient adenomas [75] and tumorigenesis in β-catenin driven cancers relay at least in part in the formation of YAP-β-catenin-TBX5 transcriptional complexes [76]. Thus, interactions between the Hippo and Wnt pathways might depend on cell type, cell context and subcellular localization. Finally, it has been shown that intrinsic signals such as energy stress, glucose metabolism, aerobic glycolysis and the mevalonate pathway can regulate YAP activity [77,78,79,80,81]. 

## 4. The Hippo-YAP Pathway in HNSCC

It is of no surprise that a signaling pathway controlling cell growth and connected to cell polarity and adhesion, cytoskeleton dynamics, cell survival factors signals as well as metabolism, is almost necessarily deregulated during cancer initiation, progression and metastasis. Different components of the Hippo pathway act as oncogenes (YAP, TEADs) or tumor suppressors (LATS1/2), and alterations in these factors have been described across different cancer types [10]. Moreover, many of the above-mentioned signals controlling Hippo-YAP activity are well-known cancer pathways [10,23]. This could explain why YAP/TAZ dependent gene expression is more widely deregulated in human cancer than might be expected by the frequency of alterations in its core components. Furthermore, it has been recently shown that through binding to chromatin readers, YAP/TAZ can heighten the expression of a specific set of genes to which cancer cells are addicted to [13,82]. Interestingly, YAP/TAZ signaling seems to be largely dispensable for the normal homeostasis of adult tissues [14,71,83,84,85,86,87], making the pathway an amenable therapeutic target in cancer. 

**Figure 2:**
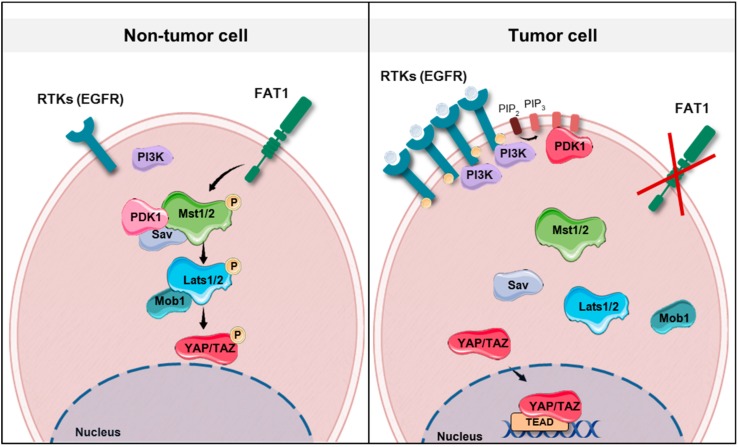
Schematic representation showing the potential molecular mechanisms involved in tumor development in the context of FAT1 (FAT atypical cadherin 1) functional loss, *EGFR* (epidermal growth factor receptor) amplification or *PIK3CA* (phosphatidylinositide 3-kinase catalytic subunit alpha) overexpression in head and neck squamous cell carcinomas (HNSCCs). Note that *PIK3CA* codes for the catalytic subunit of PI3K (phosphatidylinositide 3-kinase). In non-tumor cells, in the presence of low levels of EGFR and normal *PIK3CA* expression, PDK1 (phosphoinositide-dependent kinase 1) forms a complex with the Hippo signaling core complex promoting YAP phosphorylation. Similarly, FAT1 acts as a scaffold for Hippo kinases, thus favoring their activation. In a tumor cell, the absence of FAT1 or the presence of high levels of EGFR and increased PI3K activity, which recruits PDK1 to the cell membrane, dismantles the Hippo core complex leading to YAP dephosphorylation and its translocation to the nucleus. RTKs: receptor tyrosine kinases.

**Figure 3:**
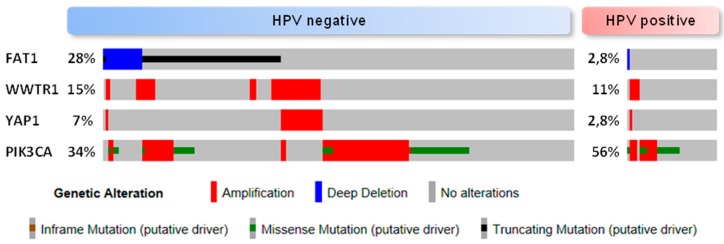
Oncoprint (cBioportal) for the HNSCC TCGA cohort [3] which includes comprehensive genomic data of human papillomavirus (HPV) negative (n = 243) and HPV positive tumors (n = 36). The alterations shown are *YAP* (Yes-associated protein) and *TAZ* (WWTR1, WW domain containing transcription regulator 1) amplification, *FAT1* inactivation (homozygous deletion and putative driver mutation) and *PIK3CA* alteration (amplification and putative driver mutation). Percentages are relative to the complete number of tumors in each dataset (HPV positive/negative).

The average frequency of alterations in the Hippo pathway across human cancer is 10% [10]. However, this figure rises to more than 90% in *IDH* mutant low grade glioblastoma, around 50% in MSI-POLE (microsatellite instability-DNA polymerase epsilon) subtypes in colorectal, stomach and endometrial tumors, and to 42% in HPV negative HNSCC [10]. HNSCC arises in different locations of the upper aerodigestive tract and one might expect that the primary site where the tumor arises could have an influence on the characteristics of the tumor, including its genetic features. However, current data [10] do not show an association between alterations in the Hippo pathway genes and different locations of HNSCC. Future research in the subject could demonstrate otherwise. Amplification in *YAP* and *TAZ* are found in 5% and 9%, respectively, of the HNSCC tumors of the TCGA Pan Cancer Atlas [10]. Additionally, two upstream regulators of this pathway are frequently altered in HNSCC, namely FAT1 and *PIK3CA*. Inactivation of *FAT1* (deletion, truncating mutations) or activation of *PIK3CA* (overexpression) are associated with YAP-dependent transcriptional activation in HNSCC [24,27]. The precise molecular mechanisms that contribute to tumor development in the context of FAT1 functional loss or *PIK3CA* overexpression are not fully understood (Figure 2). It has been shown that, in HNSCC derived cell lines, FAT1 directly associates with MST1 which favors its phosphorylation and the assembly of the Hippo kinase core complex leading to the subsequent phosphorylation of LATS1/2 and YAP [27]. Overexpression of *PIK3CA* is associated with poor outcome in HPV negative HNSCC; these tumors show YAP nuclear localization and a YAP-activation transcriptional signature [24]. Although the molecular mechanism linking PI3K and YAP in HNSCC has not been identified so far, in other epithelial cell lines activated PI3K recruits PDK1 to the plasma membrane disrupting its association with the Hippo core complex kinases and promoting YAP dephosphorylation [72,88]. The PI3K-PDK1 pathway integrates signals from fibronectin, LPA, GPCR receptors and EGFR [68]. Interestingly, despite the fact that nuclear YAP localization has been described in oropharyngeal HPV positive tumors [89], Hippo pathway alterations and in particular FAT1 inactivation or YAP amplification are not frequent events in HPV positive HNSCC [10] (Figure 3). Although further research into the role of the Hippo-YAP pathway in this tumor subtype is needed, it is tempting to speculate that other mechanisms might lead to YAP activation. For instance, *PIK3CA* alterations are the most common genetic event in HPV positive tumors [21], and it has been described that the HPV E6 oncoprotein can degrade the Hippo core complex scaffolding element Scribble [90].

In the normal oral epithelium YAP and TAZ level is generally low except for the basal layer cells that display evident nuclear YAP staining and some TAZ staining [54,91]. During hyperplasia and dysplasia cells with nuclear YAP extend beyond the basal cell population and are frequent in regions of severe dysplasia [54]. Activation of YAP/TAZ would confer these cells a proliferative advantage. There is no evidence that *YAP* and *TAZ* are significantly mutated, amplified or overexpressed in OSCC tumors [54]. *YAP* and *TAZ* expression was not associated with tumor stage or grade in some cohorts [54]. This suggests that alteration in YAP/TAZ upstream regulators (i.e., FAT1 and *PIK3CA* in HNSCC [24,27]) takes place during HNSCC tumor progression leading to the activation of these two co-transcriptional factors and their target genes [54]. At the clinical level in HNSCC, YAP and TAZ have been proposed as poor prognosis markers [24,91,92,93,94] and YAP activation has been associated with resistance to different anticancer therapies [95,96,97]. Analysis of YAP/TAZ expression signatures in OSCC cell lines indicated that YAP has a more prominent role than TAZ in the regulation of transcription, at least in this type of cancer [54]. That said, in OSCC, overexpression of TAZ has been associated with poor outcome and aggressive tumor features such as size, grade and lymph node spreading in some studies [91] while other show no association with tumor grade or stage [54]. Interestingly, the analysis of YAP/TAZ transcriptional targets in OSCC [54] revealed that increased expression of TEAD4, but not other canonical targets such as CTGF or CYR61, associated with increased tumor grade or stage in the TCGA cohort of HNSCC [54]. Additionally, TEADs can favor YAP/TAZ nuclear retention [98] thus further enhancing YAP/TAZ-TEAD4 mediated gene transcription in these tumors. A signature based on HNSCCs with *YAP* amplification and overexpression, defined a YAP-activated subgroup of tumors with worse prognosis across different HNSCC cohorts [94]. In this study, the YAP-inactivated subgroup associated with HPV positive status, which was consistent with the absence of *YAP* amplification in HPV positive HNSCC [10]. *YAP* amplification, but not high EGFR protein levels, was identified as biomarker of resistance to Cetuximab [95]. Increased YAP expression has been associated with resistance to cisplatin [96] and to radiotherapy [97]. Furthermore, TAZ depletion restores sensitivity to cisplatin in nasopharyngeal carcinoma cells [99]. 

The tumor immune microenvironment (TIME) plays an important role in many tumors including HNSCC [100]. HNSCCs with YAP amplification and/or overexpression associate with resistance to the immunotherapy agent Pembrolizumab [94]. Conversely, it has been reported than in other tumor types such as lung and melanoma, YAP induced PD-L1 expression suggesting that immunotherapy could be effective against these tumors [101,102]. There is further evidence that YAP expression in cancer cells can influence the recruitment and characteristics of the immune cells in the through the production of cytokines. YAP activity in cancer cells induces the expression of cytokines such as IL-6 (interleukin 6), CXCL5 (C-X-C motif chemokine 5), and granulocyte-macrophage colony stimulating factors that stimulate the recruitment of myeloid-derived suppressor cells (MDSCs) [103,104]. MDSCs inhibit cytotoxic T cell activity, contributing to promote an immune-suppressive tumor-tolerant microenvironment, and promote tumor angiogeneisis [105]. Immunodepletion of MDSCs reduced tumor growth in an in vivo model of oral cancer [106]. Large numbers of Tregs (regulatory T cells) are present in the HNSCC and their abundance is associated with poor prognosis [107,108]. This subset of T cells also contributes to an immunosuppressive microenvironment. In Tregs, YAP activity is required for their accumulation and suppressive function [109].

## 5. Therapeutic Opportunities for HNSCC Targeting the Hippo-YAP Pathway 

Different inhibitors have been identified to directly target YAP/TAZ, their upstream regulators or their downstream effectors. The challenge is, however, to find those more suitable for the treatment of HNSCC and to identify subgroups of patients that would benefit from these therapies. Direct inhibitors of YAP/TAZ include the drug Verteporfin and a synthetic polypeptide termed “super-TDU” designed to hamper YAP-TEAD interaction [110]. Verteporfin is an FDA-approved drug marketed under the name of Visudyne for the treatment of patients with certain serious eye conditions [111]. It has shown good results in different cancer models including liver, pancreatic, gastric and head and neck [27,112,113,114,115], and is under clinical trials for prostate and breast cancer (ClinicalTrials.gov Identifier NCT03067051 and NCT02872064, respectively). However, clinical trials are based on the photodynamic properties of the drug and Verteporfin is used there as a photosensitizer. While Verteporfin can effectively disrupt YAP/TAZ interaction with TEADs [56], other effects for this drug have been described, such as inhibition of autophagosome formation [116,117], thus making it not so suitable for use as a specific YAP/TAZ transcriptional inhibitor in cancer. Super-TDU competes with YAP in binding to TEADs and is still under preclinical development. It has been proven to be effective in gastric and colorectal cancer models [110,118]. To our knowledge, there are no reports regarding the use of this peptide in HNSCC. However, two different HNSCC cell lines (WSU-HN13 [119] and FaDu [120]) were assayed in our laboratory and Super-TDU did not show an inhibitory effect in cell growth even at nearly micromolar concentrations (unpublished observation). 

A feasible anti-tumor strategy would be to target pathways upstream or downstream of YAP/TAZ relevant for each cancer type. Statins and Dasatinib (Src inhibitor) were identified as candidate drugs to inhibit YAP/TAZ activity in cancer cells [91,121,122,123]. Although there are examples of the use of both, studies in HNSCC specifically addressing their effect on YAP/TAZ activity are scarce [91]. It is worth mentioning that an inhibitory role for simvastatin has been observed in a model of OSCC [91]; this statin was able to repress TAZ resulting in an anticancer effect [91]. Given that the PI3K-PDK1 axis mediates YAP/TAZ activation by different stimuli (EGFR, FAK, fibronectin, GPCRs) and that alterations in the *PIK3CA* gene are frequent events in HNSCCs and are associated with YAP transcriptional activation and poor outcome [24], specific PI3K inhibitors should be evaluated. Recently, inhibitors of the bromodomain and extra-terminal domain (BET) family of proteins (BRD1–4) have shown successful results in the treatment of HNSCC, including tumor models resistant to Cetuximab [124,125,126]. This brings HNSCC therapeutic options into the thriving field of epigenetics. BETs are chromatin readers and mainly recognize lysine acetylation in H3 and H4, thus influencing gene expression [127]. In the nucleus, YAP-TAZ-TEAD1 complexes interact with BRD4 and drive the expression of sets of genes involved in cancer transcriptional programs [13]. Furthermore, alterations in some components of the Hippo pathway are determinants of sensitivity to BET protein inhibitors [128]. Some BET protein inhibitors, such as Birabresib, are under clinical trials in different hematologic and solid tumors [129,130,131], but not in HNSCC. The small molecule JQ-1 specifically targets BRD4 and inhibited tumor growth and metastasis in a chemical-induced orthotropic model of HNSCC and in PDX (patient derived xenografts) [132]. Thus, although BET protein inhibitors seem a promising therapeutic strategy for the treatment of HNSCC with YAP/TAZ activation, more research is needed in the field before translating these advances into the clinical setting. 

The identification of key players in HNSCC such as FAT1 and PI3K as regulators of the Hippo-YAP pathway, as well as the activation of YAP as a relevant oncogenic mechanism in head and neck cancer opens the way for the use of different therapeutic strategies targeting this pathway in this tumor type. In particular, small-molecule inhibitors of Hippo-YAP upstream activators or inhibitors of YAP transcriptional activity are currently available in the clinical setting or are under development. Presently, research should focus on understanding the precise mechanisms of action of these drugs in the context of HNSCC using both in vivo and in vitro models. These studies will set the bases for much needed clinical trials that contribute to broaden the therapeutic options for this type of cancer. Ideally, the application of these therapies should go hand-in-hand with the identification and validation of biomarkers for this pathway, such as the abovementioned, that allow a molecularly-based stratification of patients.

## Figures and Tables

**Table 1:** Selection of the top 100 genes (by fold change gene expression) regulated by YAP and TAZ in oral squamous cell carcinoma (OSCC) cell lines [54] that are involved in different molecular processes or pathways relevant to head and neck squamous cell carcinoma (HNSCC) progression. Genes also present in the consensus Cordenonsi Yap signature [53] are also shown. Note that none of the genes shared with Cordenonsi signature increase in HNSCC with grade or stage. Gene signatures are from the MSigDB (Molecular Signature Data Base. Broad Institute). Abbreviations for these genes are shown in Supplementary Table S1.

Genes that Increase Their Expression with Tumor Grade/Stage in HNSCC					Genes Shared with Consensus YAP Signature (Cordenonsi)
Epithelial to Mesenchymal Transition (Sarrio)	Cell Cycle (REACTOME)	Nasopharyngeal Carcinoma UP (Sengupta)	Head and Neck Cancer with HPV UP (Slebos)	WNT3A Targets UP (Labbe)	
*BLM* *CDC6* *DIAPH3* *DSCC1* *DTL* *EXO1* *HELLS* *MCM6* *MYBL1*	*CCNE2* *CDC25A* *CDC6* *CENPI* *CENPK* *GINS1* *GINS2* *LMNB1* *MCM10* *MCM6* *POLE2*	*ATAD2* *CCNE2* *CENPK* *DIAPH3* *DSCC1* *DTL* *ESCO2* *FANCI* *GINS1* *HELLS* *POLE2* *RAD51AP1*	*CENPK* *FAM111B* *MCM6*	*HELLS* *MCM6*	*ANKRD1* *AXL* *CTGF* *CYR61* *DDAH1* *FSTL1* *SLIT2* *THBS1*

## Supplementary Materials

The following are available online at https://www.mdpi.com/2077-0383/8/12/2131/s1.
